# Prenylated Flavonoids with Selective Toxicity against Human Cancers

**DOI:** 10.3390/ijms24087408

**Published:** 2023-04-18

**Authors:** Tomasz Tronina, Agnieszka Bartmańska, Jarosław Popłoński, Magdalena Rychlicka, Sandra Sordon, Beata Filip-Psurska, Magdalena Milczarek, Joanna Wietrzyk, Ewa Huszcza

**Affiliations:** 1 Department of Food Chemistry and Biocatalysis, Wrocław University of Environmental and Life Sciences, C.K. Norwida 25, 50-375 Wrocław, Poland; tomasz.tronina@upwr.edu.pl (T.T.);; 2 Department of Experimental Oncology, Hirszfeld Institute of Immunology and Experimental Therapy, Weigla 12, 53-114 Wroclaw, Poland

**Keywords:** xanthohumol, aurone, antiproliferative activity, anticancer selectivity, human carcinoma

## Abstract

The antiproliferative activity of xanthohumol (**1**), a major prenylated chalcone naturally occurring in hops, and its aurone type derivative (*Z*)-6,4′-dihydroxy-4-methoxy-7-prenylaurone (**2**) were investigated. Both flavonoids, as well as cisplatin as a reference anticancer drug, were tested in vivo against ten human cancer cell lines (breast cancer (MCF-7, SK-BR-3, T47D), colon cancer (HT-29, LoVo, LoVo/Dx), prostate cancer (PC-3, Du145), lung cancer (A549) and leukemia (MV-4-11) and two normal cell lines (human lung microvascular endothelial (HLMEC)) and murine embryonic fibroblasts (BALB/3T3). Chalcone **1** and aurone **2** demonstrated potent to moderate anticancer activity against nine tested cancer cell lines (including drug-resistant ones). The antiproliferative activity of all the tested compounds against cancer and the normal cell lines was compared to determine their selectivity of action. Prenylated flavonoids, especially the semisynthetic derivative of xanthohumol (**1**), aurone **2,** were found as selective antiproliferative agents in most of the used cancer cell lines, whereas the reference drug, cisplatin, acted non-selectively. Our findings suggest that the tested flavonoids can be considered strong potential candidates for further studies in the search for effective anticancer drugs.

## 1. Introduction

Cancer is a serious group of diseases involving uncontrolled cell proliferation, which causes millions of deaths every year, making it the second leading cause of death in low- and middle-income countries [1]. Therefore, it is extremely important to prevent and protect against the development of cancer in the body. It is imperative to discover new compounds for prevention and treatment. Unfortunately, most of the substances tested, whether used in clinical applications or experimental studies, are relatively non-specific, often exerting adverse effects on normal cells. Commonly used anticancer drugs, such as doxorubicin or cisplatin, have major disadvantages, and they are toxic to both cancerous and normal cells. For this reason, small molecules with selective activity are being intensively searched for, i.e., those that would be active against cancer cells and not toxic to normal cells. Therefore, natural compounds isolated from traditional medicinal plants and their semisynthetic derivatives appear to be ideal candidates as potential anticancer agents.

Flavonoids, such as chalcones, exhibit numerous biological properties that are beneficial to human health, and most importantly, they are relatively non-toxic. One of the most biologically active natural chalcones is xanthohumol (**1**).

Xanthohumol (**1**) (3′-[3″,3″-dimethylallyl]-2′,4′,4-trihydroxy-6′-methoxychalcone), the major prenylflavonoid found in the hop (*Humulus lupulus* L.), is known for its various biological properties, such as anti-inflammatory, antioxidant, antibacterial, antifungal, antiplasmodial and antiviral properties [2,3,4]. One of the most important findings over the past 20 years was the discovery of its anticancer activity [5,6,7,8,9]. Xanthohumol (**1**) significantly suppresses the development of a wide variety of human cancer cells in vitro during the initiation, promotion and progression of carcinogenesis. Hence, it has been characterized as a “broad spectrum” cancer chemopreventive agent [2]. Despite the well-documented anticancer activity of xanthohumol (**1**), only a few papers concerning the antiproliferative activity of xanthohumol derivatives, metabolites or analogues have been published so far [10,11,12,13,14,15,16,17,18,19,20,21,22]. Our previous studies showed that having the structure of the prenylated flavonoid with non-modified prenyl moiety is crucial for the high antiproliferative activity of xanthohumol metabolites against breast cancer (MCF-7) and prostate cancer (PC-3) [15,20]. Chalcones, including dihydrochalcones, are significantly stronger anticancer agents than the related flavanones [15]. Our observations were similar to those described by Miranda [10].

One class of flavonoids is aurones, which are considered “minor flavonoids” because they naturally occur in relatively small amounts than other classes of flavonoids, such as flavones or flavanones. Aurones are most famous for their bright golden color and characteristic fluorescence that they provide to some ornamental flowers, such as sunflowers [23]. However, they are also attributed to biological properties, including anti-tumor activity. Although aurones naturally occur in small amounts, they can be obtained with relatively high yields in a cyclization reaction using chalcones as the starting material [24]. This provides opportunities to study the biological properties of non-naturally occurring aurones and compare them to analogous chalcones and flavonoids belonging to other classes so that the effect of the flavonoid skeleton on the biological activity can be determined.

In continuation of our research on xanthohumol derivatives as potential therapeutic agents, in our previous studies, we synthesized an “aurone type” derivative, which was the starting material for obtaining glycosylated derivatives, and then the influence of the skeleton type of the prenylated flavonoids and the presence of sugar moiety in the flavonoid structure on the binding to human serum albumin and the inhibition of the cyclooxygenases COX-1 and COX-2 were investigated [25]. In this paper, xanthohumol (**1**) and its aurone derivative (**2**), as well as cisplatin as a reference anticancer drug, were examined against ten human cancer and two normal cell lines and then compared to determine their selectivity of action. The studies demonstrate how the modification of a prenylated chalcone into its aurone derivative may affect anticancer activity and selectivity.

To the best of our knowledge, the antiproliferative activities of xanthohumol, an aurone-type derivative (**2**), towards human cancers as well as normal cells have not been published so far.

## 2. Results

**Figure 1:**
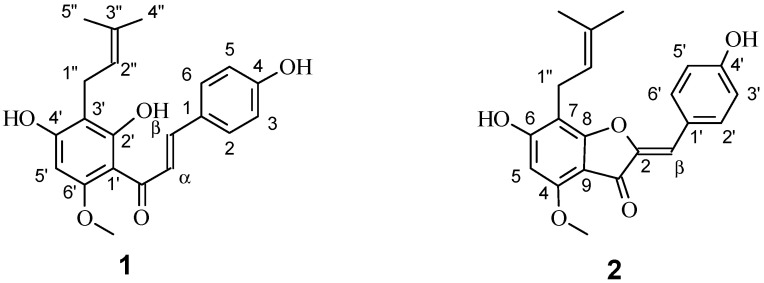
Structures of xanthohumol (**1**) and its semisynthetic derivative (*Z*)-6,4′-dihydroxy-4-methoxy-7-prenylaurone (**2**).

Xanthohumol (**1**), its aurone (**2**) (Figure 1) and cisplatin were evaluated for their antiproliferative activity towards the following human cancer cell lines: breast (MCF-7, SK-BR-3, T47D), prostate (PC-3, Du145), colon (HT-29, LoVo, LoVoDx), lung (A549) and leukemia (MV-4-11).

**Figure 2:**
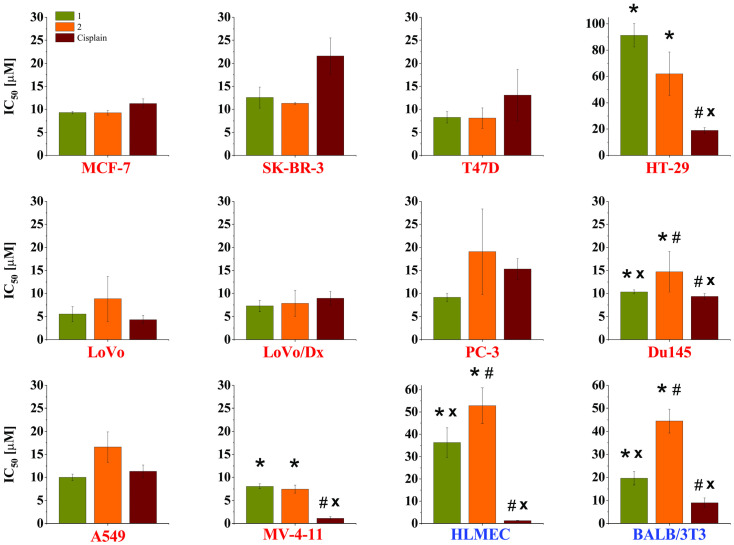
In vitro antiproliferative activity of xanthohumol (**1**) and aurone (**2**) against human cancer cell lines (breast cancer (MCF-7, SK-BR-3, T47D), colon cancer (HT-29, LoVo, LoVo/Dx), prostate cancer (PC-3, Du145), lung cancer (A549) and leukemia (MV-4-11)) and two normal cell lines (human lung microvascular endothelial (HLMEC) and murine embryonic fibroblasts (BALB/3T3)). Red Labels—cancerous cell lines, Blue Labels—normal cell lines. Data represent the mean values of at least three independent experiments (*n* = 3) ± SD. X—value obtained in a single assay, in other three independently repeated experiments not active at the concentration applied. Statistically significant at * *p* value < 0.05 vs. cisplatin; **#** *p* value < 0.05 vs. **1** (xanthohumol (**1**)); x *p* value < 0.05 vs. **2** (aurone (**2**)). Numeric values of IC_50_ of tested compounds towards examined cell lines are presented in Table S1 in Supplementary Materials.

Additionally, the cytotoxicity of these compounds was also tested against normal cell lines: murine embryonic fibroblasts (BALB/3T3) and human lung microvascular endothelium (HLMEC). One of the used cancer cell lines was resistant to doxorubicin (LoVo/Dx). The concentrations of the tested compounds (µM) at which 50% cell growth inhibition was observed are summarized in Figure 2.

Both xanthohumol (**1**) and its aurone (**2**) showed potent (MCF-7, T47D, LoVo, LoVo/Dx, MV-4-11) to moderate (SK-BR-3, PC-3, Du145, A549) antiproliferative activity against the examined cancer cell lines, except colon cancer cells (HT-29), in which case the compounds were rather weakly active (IC_50_ > 50 µM). Xanthohumol (**1**) and aurone (**2**), as well as cisplatin, displayed no significant differences in their antiproliferative effect towards the MCF-7, SK-BR-3, T47D, LoVo, LoVo/Dx, PC-3 and A549 cancer cell lines (*p* > 0.05). Among all the used cancer cell lines, cisplatin, which is commonly used as a drug in anticancer therapy, showed significantly higher activity (*p* < 0.05) only in the case of two cancer cell lines: colon cancer (HT-29) and leukemia (MV-4-11). In the case of the colon cancer cell line, both flavonoids showed relatively low antiproliferative activity (91.31 ± 8.92 µM and 62.09 ± 16.52 µM), whereas cisplatin was 4.8-fold and 3.3-fold more active, respectively. In the case of the MV-4-11 leukemia cancer cell line, both flavonoids displayed high antiproliferative activities (8.07 ± 0.52 µM and 7.45 ± 0.87 µM, respectively). However, cisplatin was significantly more active (7.3-fold and 6.7-fold, respectively). The significant differences in the antiproliferative activities between all the tested compounds (*p* < 0.05) were recorded. In the case of the prostate cancer cell line (Du145), cisplatin was the most active, xanthohumol (**1**) was only slightly less active and aurone (**2**), with a value of IC_50_ = 14.71 ± 4.42 µM, was the least effective at inhibiting the growth of the prostate cancer line. The obtained results of the antiproliferative activity of the tested flavonoids against the human cancer cell lines often revealed no statistical difference in activity compared to cisplatin (among the ten cancer cell lines tested, as many as seven displayed no difference in activity (*p* > 0.05)), which may indicate that both xanthohumol **1** and its aurone **2** may be potential candidates as compounds for use in anticancer therapy.

The selective effect of a drug can be expressed by the selectivity index (SI), which is determined by comparing the cytotoxic activity of each compound against each cancer cell line with the activity against a normal cell line. The SI was calculated as the IC_50_ ratio for a normal cell line (SI_A_—HLMEC; SI_B_—BALB/3T3) to the IC_50_ value for the corresponding tumor cell line using the following equation:

(1) $$\mathrm{SI}=\frac{{\mathrm{IC}}_{50}\;\mathrm{for}\;\mathrm{normal}\;\mathrm{cell}\;\mathrm{line}}{{\mathrm{IC}}_{50}\;\mathrm{for}\;\mathrm{cancerous}\;\mathrm{cell}\;\mathrm{line}}$$

**Figure 3:**
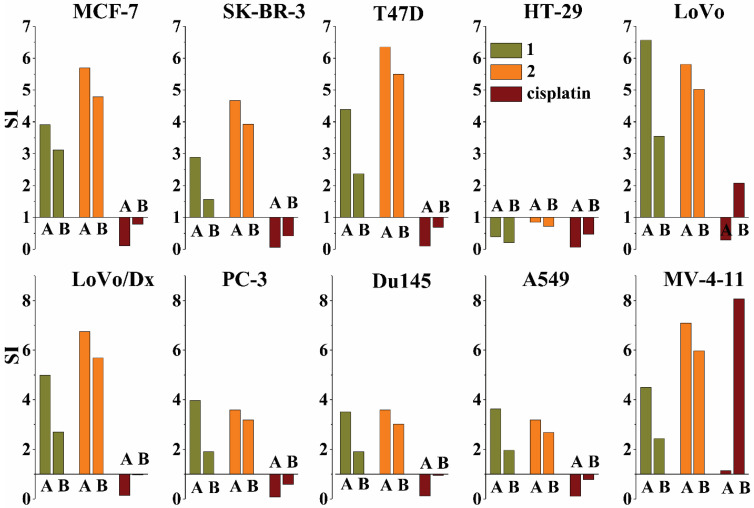
The selectivity indexes (SI) that represent IC_50_ for normal cell line/IC_50_ for cancerous cell line. Selectivity index (SI) was calculated for each compound using the following formula: A = SI_A_ = IC_50_ for normal cell line (HLMEC)/IC_50_ for respective cancerous cell line (breast cancer (MCF-7, SK-BR-3, T47D), colon cancer (HT-29, LoVo, LoVo/Dx), prostate cancer (PC-3, Du145), lung cancer (A549) and leukemia (MV-4-11)), as indicated on each plot; B = SI_B_ = IC_50_ for normal cell line (BALB/3T3)/IC_50_ for respective cancerous cell line, as indicated on each plot. SI > 1.0 indicates a drug with efficacy against tumor cells greater than the toxicity towards normal cells. SI < 1.0 indicates nonselective action. Numeric, calculated values of SI_A_ and SI_B_ of tested compounds are presented in Table S2 in Supplementary Materials.

The SI values higher than 1.00 indicate that the tested compound exhibited higher selectivity towards cancer cells than normal ones [26]. The selectivity indexes of the tested compounds are shown in Figure 3.

Due to the different antiproliferative activities of the tested compounds against two normal cell lines, human (HLMEC) and murine (BALB/3T3), the values of the selectivity indexes SIA and SIB for a given compound against a respective cancer line differ. However, without a doubt, the calculated selectivity indexes of the tested compounds clearly indicate that both tested flavonoids show high selectivity of action against all the tested cancer cell lines except colon cancer (HT-29), while cisplatin was selective only for colon cancer (LoVo cell line) and leukemia (MV-4-11 cell line). However, this result is not clear, since the selective action against the above-mentioned cancers is confirmed only by the selectivity indexes calculated for the normal murine embryonic fibroblast (BALB3/T3), SI_B_, whereas the selective indexes for human lung microvascular endothelium (HLMEC), SI_B,_ shows nonselective action of cisplatin against all the tested human cancer cell lines. These data confirm the well-known fact that many of the commonly used chemotherapeutics, including cisplatin, are toxic not only to malignant cells but also to normal cells.

The most selective among of all the investigated compounds was aurone (**2**), which exhibited very high selectivity towards all the breast cancer cell lines tested (MCF-7, SK-BR-3, T47D: SI_A_ = 4.67–5.69; SI_B_ = 3.93–5.50) and the colon cancer lines LoVo and LoVo/Dx (SI_A_ = 5.97 and 7.09; SI_B_ = 5.68 and 5.97, respectively). These results may be very interesting in the context of possible future applications of this compound, due to the resistance of the LoVo/Dx line to the commonly used anticancer drug doxorubicin. In the case of both prostate cancers (PC-3, Du145), as well as lung cancer (A549), aurone **2** was as selective as xanthohumol (**1**) considering the values of SI_A_ and slightly more selective when comparing the antiproliferative activity against cancer cell lines to the activity towards normal murine embryonic fibroblasts cells (SI_B_). The selectivity of both flavonoids against the above-mentioned cancer cell lines can be considered high (the values of both selectivity indexes close to 2.0 or greater). Very high and high selectivity of the tested prenylated flavonoids towards most of the cancer lines used indicates that both compounds can be recognized as potent and selective potential anticancer agents. Moreover, the higher selectivity of aurone (**2**) in comparison to xanthohumol (**1**) clearly shows that the modification of the flavonoid skeleton, prenylated chalcone, into its aurone derivative significantly increases the selectivity against almost all of the used cancer cell lines. 

## 3. Discussion

Commonly used anticancer drugs, such as doxorubicin and cisplatin, have a major drawback in that they are toxic to both cancerous and normal cells. The nonselective nature of anticancer drugs represents a very serious problem for cancer therapy. Therefore, the selectivity of cytotoxic agents is an important pharmaceutical parameter that facilitates the estimation of the chances for future clinical development of potential anticancer drugs. Hence, to solve the problem regarding the nonselective action of anticancer drugs, the search is on for new, effective and safe pharmaceuticals. Thus, modern screening of anticancer drugs for chemotherapy should aim not only at high anticancer activity but also at identifying agents that mostly selectively kill cancer cells. 

Natural dietary compounds, such as flavonoids and synthetic pharmacological agents, can arrest and/or reverse carcinogenesis—this process is often called chemoprevention. Recently, there has been much research focused on the biological activities of prenylated flavonoids, which very often exhibit stronger properties than those of non-prenyl parent flavonoids [27,28,29,30]. Xanthohumol (**1**), a prenylated chalcone naturally occurring in hops, is known for its various biological activities, including anticancer activity [2,4,31]. Research showed that xanthohumol (**1**) inhibited the proliferation, migration and invasion, as well as induced apoptosis and cell cycle arrest, in many types of cancer-inhibiting carcinogenesis and metastasis [2,5,6,9]. Multiple crucial pathways and signaling molecules are involved in the anticancer activity of xanthohumol (**1**), including Akt, NF-kB, ROS and ERK1/2 [5,6]. Studies showed that xanthohumol (**1**) can also enhance apoptosis induced by TRAIL in cancer cells [32,33,34]. Despite its potent antiproliferative activity and potential selective action against numerous cancers, the use of xanthohumol (**1**) raises concerns due to its conversion into the most potent phytoestrogen known, 8-prenylnaringenin (8-PN), which may promote the growth of estrogen-dependent cancers [35].

**Figure 4:**
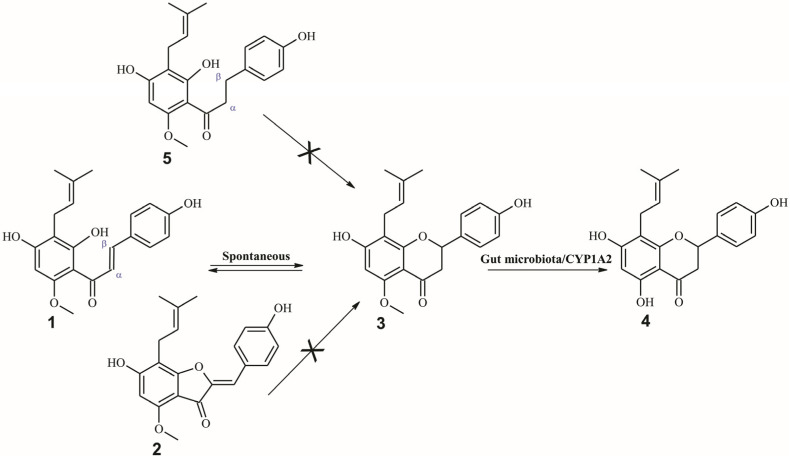
Metabolic conversion of xanthohumol (**1**) to 8-prenylnaringenin (**4**) via spontaneous cyclization to isoxanthohumol (**3**). α,β-dihydroxanthohumol (**5**) and aurone (**2**), by their inability to cyclize to isoxanthohumol (**3**), do not have the ability to convert to 8-prenylnaringenin.

This two-step reaction involves the spontaneous isomerization of xanthohumol (**1**) to isoxanthohumol (**3**) and *O*-demethylation, catalyzed by the host’s cytochrome P450 enzyme (CYP1A2) and gut microbiota, resulting in the formation of 8-prenylnaringenin (**4**) (Figure 4) [36,37,38]. The crucial element in the process of the isomerization of the chalcone–flavanone type is the presence of an α,β-unsaturated carbonyl moiety that enables intramolecular Michael addition and forms flavanones from chalcones. Therefore, xanthohumol derivatives that would exhibit strong biological properties and would not be converted to flavanones, including 8-prenylnaringenin (**4**), are strongly sought. 

Modification of the chalcone to the dihydrochalcone skeleton by the hydrogenation of the α,β-olefin bond counteracts spontaneous chalcone—flavanone isomerization. Thus, the saturation of the double bond at the α,β-position in xanthohumol (**1**) to α,β-dihydroxanthohumol (**5**) prevents isomerization to isoxanthohumol (**3**) and, therefore, conversion to the potentially dangerous 8-prenylnaringenin (Figure 4). Our previous studies showed that α,β-dihydroxanthohumol (**5**) might be as effective an antiproliferative agent against human breast cancer cell lines (MDA-MB-231,T-47D and MCF-7) and an ovarian cancer cell line (A2780) as xanthohumol (**1**). Both prenylated flavonoids exhibited a comparable growth-inhibiting effect, whereas structural modifications, such as the isomerization of xanthohumol (**1**) to isoxanthohumol (**3**) or the demethylation of isoxanthohumol (**3**) to 8-prenylnaringenin (**4**), led to a reduction in the cytostatic activity towards the cancer cell lines tested [19]. The aim of the present study was to demonstrate how the modification of chalcone to an aurone derivative, which also does not have the ability to form 8-prenylnaringenin (**4**) in the body, can affect the growth inhibition and selectivity against human cancer cell lines in vitro. Therefore, the antiproliferative properties and selectivity of the aurone derivative of xanthohumol (**2**) against ten human cancer cell lines in comparison to xanthohumol (**1**) and a reference, cisplatin (a drug commonly used in anticancer treatment), was investigated. Our studies have shown that the modification of xanthohumol (**1**) to an aurone derivative does not adversely affect the antiproliferative activity against the human cancer cell lines tested. Both xanthohumol (**1**) and its aurone (**2**) showed potent to moderate antiproliferative activity against the examined cancer cell lines. This is a remarkable finding since modifications of xanthohumol (**1**) to other analogous flavonoids with a flavanone or flavone skeleton reduce its potent anticancer activity [19,20]. In addition, the modification of xanthohumol (**1**) into an aurone derivative (**2**) significantly (*p* < 0.05) reduced the cytotoxic effect against normal cells: 1.5-fold in the case of the human lung microvascular endothelial (HLMEC) cell line and 2.3-fold in the case of the murine embryonic fibroblasts (BALB/3T3) cell line, respectively. The comparable antiproliferative activities of flavonoids **1** and **2**, as well as the significantly lower cytotoxicity of aurone **2** towards normal cells lines, makes this compound a more selective anticancer agent than xanthohumol (**1**) against breast cancers (MCF-7, Sk-Br-3, T-47D), doxorubicin-resistant colon cancer (LoVo/Dx) and leukemia (MV-4-11), whereas in the case of prostate cancer (PC-3, Du145) and lung cancer (A549), aurone **2** exhibited a comparable selectivity of action as xanthohumol (**1**). Since aurone **2** is not able to be transformed into potentially dangerous 8-prenylnaringenin, as happens in the case of xanthohumol (**1**), the obtained results may be very interesting in the context of possible future applications of this compound as a potential anticancer drug, including estrogen-dependent ones.

## 4. Materials and Methods

### 4.1. Chemicals

Mercury (II) acetate (ACS reagent, ≥99.0%), sodium bicarbonate, magnesium sulfate, L-glutamine, sodium pyruvate, amino acids, doxorubicin, insulin, sulforhodamine B, 3-(4,5-dimethylthiazol-2-yl)-2,5-diphenyl tetrazolium bromide, Tris base, hydrochloric acid, acetic acid, formic acid, dimethylformamide, sodium dodecyl sulfate and dimethyl sulfoxide-d_6_ were purchased from Sigma-Aldrich Chemie GmbH, Taufkirchen, Germany. Streptomycin and penicillin were purchased from Polfa-Tarchomin, Warsaw, Poland, and trichloroacetic acid was purchased from Avantor Performance Materials, Poland. DMEM and Eagle media (both from IIET, Wrocław, Poland), RPMI 1640 and Opti-MEM media were purchased from Gibco, Scotland, UK. All the solvents used for the syntheses and purification were of analytical grade, and those used for HPLC analysis were of HPLC grade (Sigma-Aldrich Chemie GmbH, Germany).

### 4.2. General Experimental Procedure

Column chromatography was performed using Sephadex LH-20 (GE Healthcare, Chicago, IL, USA) or silica gel 60 (230–400 mesh ASTM, Fluka) with the solvent mixtures specified in each experiment. The HPLC analyses were performed on a Waters 2695 Alliance instrument equipped with a Waters 2996 photodiode array detector (detection at wavelengths within the range of 200 to 600 nm) and using an Agilent Zorbax XDB C-18 analytical HPLC column (4.6 mm × 250 mm, 5 µm particle size). The elution was achieved at a flow rate of 1 mL/min; eluent A: 0.05% formic acid in redistilled water; eluent B: 0.05% formic acid in methanol, using the following elution program: 0–3 min isocratic A:B (55:45 *v*/*v*), 3–32 min linear gradient from A:B (55:45 *v*/*v*) to A:B (5:95 *v*/*v*), 32–37 min isocratic A:B (5:95 *v*/*v*), 37–39 min linear gradient from A:B (5:95 *v*/*v*) to A:B (55:45 *v*/*v*), with 10 min of re-equilibration of the column before the next run. High-resolution ESI-MS spectra were obtained on a Bruker micrOTOF-Q spectrometer. The direct infusion ESI-MS parameters were as follows: The mass spectrometer was operated in negative ion mode with the potential between the spray needle and the orifice at 4.5 kV, a nebulizer pressure of 0.4 bar and a drying gas flow rate of 4 L/min at 200 °C. The sample flow rate was 180 μL/min. Ionization mass spectra were collected at the range *m*/*z* 150–3000. The instrument was calibrated with an Agilent electrospray calibration solution (ESI-L low concentration Tuning Mix—Agilent Technologies, Santa Clara, CA, USA, Agilent Product Number: G1969-85000) that was diluted with acetonitrile. NMR spectra (^1^H NMR, ^13^C NMR, DEPT 135°, ^1^H–^1^H NMR (COSY), ^1^H–^13^C NMR (HSQC)) were recorded at 600 and 151 MHz on a DRX Bruker Avance instrument using DMSO-*d6* as a solvent. The chemical shifts are provided in parts per million (ppm) (δ relative to residual solvent peak for ^1^H and ^13^C). UV spectra were collected on a Cintra 303 Spectrophotometer (GBC) in methanol.

### 4.3. Materials

#### 4.3.1. Xanthohumol (**1**)

Xanthohumol (3′-[3″,3″-dimethylallyl]-2′,4′,4-trihydroxy-6′-methoxychalcone) (**1**) was isolated from supercritical CO_2_ extracted hops (‘Marynka’, crop 2011) obtained from Łukasiewicz—Institute of New Chemical Syntheses (Puławy, Poland). The method of isolation is described in the Supplementary Materials (Page S2).

#### 4.3.2. (*Z*)-6,4′-Dihydroxy-4-methoxy-7-mprenylaurone (**2**)

(*Z*)-6,4′-dihydroxy-4-methoxy-7-prenylaurone (**2**) was prepared from xanthohumol (**1**) according to the method described in our previous studies using mercury (II) acetate as a catalyst of the reaction [25]. The method of synthesis is described in the Supplementary Materials (Figure S1, Pages S2–S3).

The NMR, mass spectroscopy and spectrophotometry data of xanthohumol (**1**) and (*Z*)-6,4′-dihydroxy-4-methoxy-7-prenylaurone (**2**) are available in the Supplementary Materials (Figures S2–S7).

### 4.4. Antiproliferative Assay In Vitro

#### 4.4.1. Cells

The following established in vitro cell lines were applied: human breast adenocarcinoma (MCF-7 and SK-BR-3), human breast ductal carcinoma (T47D), human prostate adenocarcinoma (PC-3), human prostate carcinoma (Du145), human colorectal adenocarcinoma (HT-29 and LoVo), doxorubicin-resistant human colorectal adenocarcinoma (LoVo/Dx), human myelomonocytic leukemia (MV-4-11), human lung adenocarcinoma (A549), normal murine embryonic fibroblasts (BALB/3T3) and human lung microvascular endothelial (HLMEC). All the cell lines were obtained from the American Type Culture Collection (Rockville, MD, USA) except for LoVo and LoVo/DX, which were received from the Technical University of Gdańsk, Poland, courtesy of Prof. E. Borowski. All the cell lines were maintained in the Institute of Immunology and Experimental Therapy, Wroclaw, Poland. The cells were cultured in the following media: DMEM medium (BALB/3T3), Eagle medium (MCF-7, SK-BR-3, Du145), RPMI medium (HLMEC, MV-4-11) or RPMI 1640 + Opti-MEM (1:1) medium (A549, T47D, LoVo, LoVo/Dx, PC-3, HT-29). The DMEM medium was supplemented with 2 mM L-glutamine and 10% fetal bovine serum. The Eagle medium was supplemented with 4 mM L-glutamine, 10% fetal bovine serum, 1 mM sodium pyruvate for Du145 cells or 2 mM L-glutamine, 1% amino acids and 0.8 mg L^−1^ of insulin for MCF-7 and SK-BR-3. The RPMI medium was supplemented with 2 mM L-glutamine, 1 mM sodium pyruvate and 10% fetal bovine serum. The RPMI 1640 + Opti-MEM (1:1) medium was supplemented with 2 mM L-glutamine, 5% fetal bovine serum (PC-3, A549) and 0.8 mg L^−1^ insulin (T47D) or 1 mM sodium pyruvate (HT-29, LoVo, LoVoDx) and 0.1 ug/mL doxorubicin (LoVo/Dx). The media contained antibiotics: 100 µg mL^−1^ streptomycin and 100 U mL^−1^ penicillin. All the cell lines were incubated at 37 ºC in a humid atmosphere saturated with 5% CO_2_. 

#### 4.4.2. Assays

##### SRB Assay

The details of this technique were described by Skehan et al. [39]. After 72 h of incubation with the tested compounds, the cells were fixed in situ by gently adding cold 50% trichloroacetic acid—TCA (50 µL per well). The plates were incubated at 4 °C for 1 h and then washed five times with tap water and air-dried. Next, the cellular material fixed with TCA was stained by adding 50 µL of 0.4% sulforhodamine B dissolved in 1% acetic acid to each well and incubating the wells for 30 min at room temperature. The unbound dye was removed by washing the plates five times with 1% acetic acid, whereas the stain bound to the cells was extracted with 150 µL of 10 mM unbuffered Tris base for determination of the optical density at a 540 nm wavelength in a computer-interfaced, 96-well microtiter plate reader–Multiskan FC photometer (Thermo Scientific, Waltham, MA, USA). The background optical density was measured in the wells filled with culture medium, without the cells. 

##### MTT Assay

The details of this technique were described by Antoszczak et al. [40]. The proliferation inhibition of human myelomonocytic leukemia (MV-4-11) by the tested compounds was measured using the MTT assay. Thus, 20 µL of 3-(4,5-dimethylthiazol-2-yl)-2,5-diphenyl tetrazolium bromide solution was added to each well. Then, the plates were incubated for 4 h in a cell incubator to allow the cells to metabolize the yellow MTT to blue formazan. Next, 80 µL of lysing mixture (consisting of 67.5 g sodium dodecyl sulfate, 225 mL dimethylformamide and 275 mL distilled water) was added to each well. The plates were incubated for 24 h for the formazan crystals to be released from the cells and dissolved, and then the absorbance of each well was read on a 96-well microtiter plate reader (Multiskan FC photometer (Thermo Scientific)) at a 570 nm wavelength.

Twenty-four hours before incubation with the tested compounds, the cells were seeded in 96-well plates (Sarstedt, Nümbrecht, Germany) at a density of 10^4^ cells per well (10^3^ cells per well for HLMEC) in 100 µL of culture medium. The analysis of the antiproliferative activity of the compounds was performed using sulforhothamine B (SRB) (for all the adherent cells) or 3-(4,5-dimethylthiazol-2-yl)-2,5-diphenyl tetrazolium bromide (MTT) (for the leukemia cells—MV-4-11). The details of this technique have been previously described by Skehan et al. [39] and Antoszak et al. [40]. An assay was performed after 72 h of exposure at varying concentrations (from 0.1 to 100 µg mL^−1^) of xanthohumol (**1**), aurone (**2**) and the reference drug cisplatin. Each compound at each concentration was tested in a single experiment, which was repeated at least 3 times. DMSO, which was used as a solvent in a dilution corresponding to its highest concentration applied to the tested compounds, did not exert any inhibitory effect on cell proliferation. The results were calculated as the IC_50_ (μM) (half maximal inhibitory concentration), i.e., the concentration of the tested agent that inhibits the proliferation of the cancer cell population by half [41]. The IC_50_ values and the mean values ± SD calculated for each experiment separately using GraphPad Prism software (GraphPad Software 9.1.0, San Diego, CA, USA) are presented in Table S1 in the Supplementary Materials.

### 4.5. Statistical Analysis

The data are presented as the mean value ± standard error of the mean (SD). Statistical analysis was performed with the one-way ANOVA with Tukey’s post hoc test (Statistica Software 13.0, StatSoft). The significance was accepted at a *p* value < 0.05.

## 5. Conclusions

Plants are a great source of many natural compounds that have potential in the prevention and treatment of numerous diseases, including cancer. The major prenylated flavonoid of hops, xanthohumol (**1**), has remarkable biological properties. The anticancer activity of xanthohumol (**1**) has been the subject of studies for more than 20 years. It is known that xanthohumol (**1**) may induce cell cycle arrest and apoptosis in cancer cells. Numerous cell signaling pathways, such as NF-κB, Akt, ERK, ROS, STAT3, Notch and AMPK, are involved in the anticancer activity of this prenylated chalcone [5,6]. Potent biological properties make xanthohumol (**1**) an excellent starting material for obtaining other biologically active compounds. Our studies have shown that both xanthohumol (**1**) and its semisynthetic aurone derivative (**2**) have significant antiproliferative activity against nine out of ten cancer cell lines, including doxorubicin-resistant human colon cancer (LoVo/Dx). Moreover, they displayed much higher cytotoxicity against almost all the tested cancer cell lines compared to the normal cell lines. 

The higher selectivity of aurone (**2**) in comparison to xanthohumol (**1**) clearly shows that the chalcone–aurone type modification of the flavonoid skeleton in prenylated flavonoids may significantly increase the selectivity of action against tumor cell lines in vitro. In addition, aurone **2** is unable to convert into the potentially dangerous 8-prenylnaringenin (**4**) in the body, which could be a major advantage for its use over xanthohumol (**1**) in the context of possible future applications as a potential anticancer drug, including estrogen-dependent ones. Nevertheless, it is crucial to conduct additional research into the mechanisms of action of aurone **2** to confirm its therapeutic potential and possible use in future cancer treatment.

Our studies have shown that xanthohumol (**1**) and its aurone derivative (**2**) are potent and selective antiproliferative agents. However, further research is required to determine the molecular mechanisms of action and confirm the obtained results using in vivo cancer models.

## Data Availability

Not applicable.

## Supplementary Materials

The following supporting information can be downloaded at https://www.mdpi.com/article/10.3390/ijms24087408/s1. References [42,43,44,45] are cited in the supplementary materials.
