# Supplementary material for: Prenylated Flavonoids with Selective Toxicity against Human Cancers
Source: Int J Mol Sci. 2023 Apr 18;24(8):7408. doi: 10.3390/ijms24087408 (PMC10138577; doi:10.3390/ijms24087408)
Supplement: Supplementary file 1 [file ijms-24-07408-s001.zip › Fin.Proof_Supplementary materials - Prenylated flavonoids with selective toxicity against human cancers.pdf]

Supplementary data

Prenylated flavonoids with selective toxicity against human cancers

Tomasz Tronina[a], Agnieszka Bartmańska[a(*)], Jarosław Popłoński[a], Magdalena Rychlicka[a], Sandra Sordon[a], Beata Filip-Psurska[b], Magdalena Milczarek[b], Joanna Wietrzyk[b] and Ewa Huszcza[a]

[a] Department of Food Chemistry and Biocatalysis, Wrocław University of Environmental and Life Sciences, Norwida 25, 50-375 Wrocław, Poland

[b] Department of Experimental Oncology, Hirszfeld Institute of Immunology and Experimental Therapy, Weigla 12, 53-114 Wroclaw, Poland

Table of Contents: **Page**

Table of Contents:	Page
**Isolation of Xanthohumol (1)**	**S2**
**Synthesis of (*Z*)-6,4'-dihydroxy-4-methoxy-7-prenylaurone (2)**	**S2-S3**
**UV spectra of xanthohumol (1) and its aurone (2)**	**S4**
**HRMS spectrum of (*Z*)-6,4'-dihydroxy-4-methoxy-7-prenylaurone (2)**	**S4**
**NMR Spectra**	**S5-S7**
^{1}H NMR spectra of xanthohumol (1) and its aurone (2)	S5
^{13}C NMR spectra of xanthohumol (1) and its aurone (2)	S6
^{1}H–^{1}H NMR (COSY) spectrum of (Z)-6,4'-dihydroxy-4-methoxy-7-prenylaurone (2)	S7
^{1}H– ^{13}C NMR (HSQC) spectrum of (Z)-6,4'-dihydroxy-4-methoxy-7-prenylaurone (2)	S7
**Table S1. *In vitro* antiproliferative activity of xanthohumol (1) and aurone (2) against human cancer and normal human and murine cell lines**	**S8**
**Table S2. The selectivity index (SI) which represents IC_{50} for normal cell line/IC_{50} for cancerous cell line**	**S9**
**References:**	**S9**

Isolation of Xanthohumol (1)

Xanthohumol (3'-[3",3"-dimethylallyl]-2',4',4-trihydroxy-6'-methoxychalcone) (**1**) was isolated from supercritical CO_2 extracted hops ('Marynka', crop 2011), obtained from Łukasiewicz – Institute of New Chemical Syntheses (Puławy, Poland). 100 g of spent hops were extracted for 2 hours at room temperature with 1L of ethyl acetate. The extract was filtered and the solvent evaporated off then chromatographed over Sephadex LH-20 with methanol as eluent. The fractions containing xanthohumol were collected, concentrated in vacuo and purified by column chromatography on silica gel 60 using chloroform:methanol (20:1 v/v) as the eluent. The obtained crude product was purified by column chromatography on silica gel 60 using methylene chloride:diethyl ether:hexane:formic acid (200:40:28:1 v/v) as the eluent to give pure xanthohumol (**1**) with yield 370 mg (0.37%) and purity > 98% as yellow-orange crystals. Spectral data of **1** were in agreement with the literature [1-3].

Xanthohumol (1): Yellow-orange crystals. ^{1}H NMR (600 MHz, DMSO-d_6) δ_H: 1.61 (3H, s, H-5"), 1.70 (3H, s, H-4"), 3.13 (2H, d, J = 7,1 Hz, H-1"), 3.87 (3H, s, C6'O-CH_3), 5.14 (1H, m, H-2"), 6.08 (1H, s, H-5'), 6.84 (1H, m, H-3 and H-5), 7.58 (1H, m, H-2 and H-6), 7.67 (1H, d, J = 15,6 Hz, H-β), 7.77 (1H, d, J = 15,6 Hz, H-α), and 14.69 (C2'-OH). ^{13}C NMR (151 MHz, DMSO-d_6) δ_C: 17.7 (C-4"), 21.1 (C-1"), 25.5 (C-5"), 55.8 (C6'O-CH_3), 91.0 (C-5'), 104.6 (C-1'), 107.4 (C-3'), 116.0 (C-3 and C-5), 123.1 (C-2"), 123.8 (C-α), 126.1 (C-1), 130.0 (C-3"), 130.5 (C-2 and C-6), 142.6 (C-β), 160.0 (C-4), 160.6 (C-6'), 162.4 (C-4'), 164.7 (C-2'), and 191.7 (C=O); HR ESI-MS/: *m/z* calculated for $C_{21}H_{22}O_5$ - H ($[M-H]^-$): 353.1395. Found 353.1414 $[M-H]^-$. UV (MeOH) λ_{max}: 368.1 nm.

Synthesis of (*Z*)-6,4'-dihydroxy-4-methoxy-7-prenylaurone (2)

(*Z*)-6,4'-dihydroxy-4-methoxy-7-prenylaurone (**2**) has been prepared from xanthohumol (**1**) according to the method described in our previous studies using mercury (II) acetate as catalyst of reaction [4].

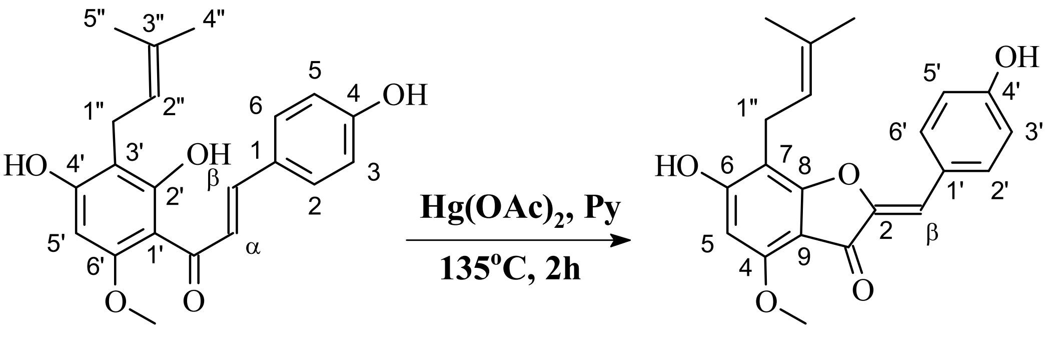

1 **2**

Figure S1. Synthesis of (Z)-6,4'-dihydroxy-4-methoxy-7-prenylaurone (**2**) from xanthohumol (**1**)

Xanthohumol (**1**) (354.4 mg, 1 mmol) was dissolved in 10 mL of a pyridine solution of mercury acetate (319 mg, 1 mmol), at room temperature. Then the mixture was refluxed for 2h and after cooling down, poured into ice-cold water (70 mL) and acidified with ice-cold 10% HCl. The mixture was extracted with ice-cold ethyl acetate (three times) The combined fractions were washed with ice-cold $NaHCO_3$ solution (three times), and ice-cold distilled water (three times), then dried over anhydrous $MgSO_4$, filtered and evaporated under vacuum to give 423 mg of crude *Z*-aurone (**2**). The product was purified by column chromatography on silica gel 60 with a mixture of methylene chloride:acetonitrile:formic acid (140:25:1 v/v) as the eluent. (*Z*)-6,4'-dihydroxy-4-methoxy-7-prenylaurone (**2**) was obtained (yield 64.8%) with over 98% of purity (according to HPLC). To avoid light-induced isomerization of *Z*-aurones to *E*-aurones [5, 6] all the procedures of synthesis and purification were performed in the dark.

(*Z*)-6,4'-dihydroxy-4-methoxy-7-prenylaurone (2): Orange crystals, yield 64.8 % (228.3 mg). ^{1}H NMR (300 MHz, DMSO-d_6) δ_H: 1.65 (3H, s, H-5"), 1.80 (3H, s, H-4"), 3.33 (2H, m H-1"), 3.81 (3H, s, C4O-CH_3), 5.25 (1H, m, H-2"), 6.23 (1H, s, H-5), 6.58 (1H, s, H-β), 6.85 (2H, m, H-3'and H-5'), and 7.78 (2H, m, H-2' and H-6'). ^{13}C NMR (150 MHz, DMSO-d_6) δ_C: 17.6 (C-4"), 21.2 (C-1"), 25.5 (C-5"), 55.6 (C4O-CH3), 94.0 (C-5), 103.0 (C-9), 103.9 (C-7), 109.5 (C-β), 115.9 (C-3' and C-5'), 122.0 (C-2"), 123.4 (C-1'), 131.0 (C-3"), 132.8 (C-2" and C-6"), 146.0 (C-2), 157.0 (C-4), 158.9 (C-4'), 164.8 (C-8), 165.2 (C-6), and 179.0 (C=O). HR ESI-MS: *m/z* calculated for $C_{21}H_{20}O_5$ - H: 351.1238 ([M- H]$^-$). Found 351.1258 [M- H]$^-$. UV (MeOH) λ_{max}: 337.1, 399.6 nm.

Figure S2: *UV spectra of: **A** xanthohumol (**1**), **B** its aurone (**2**)*

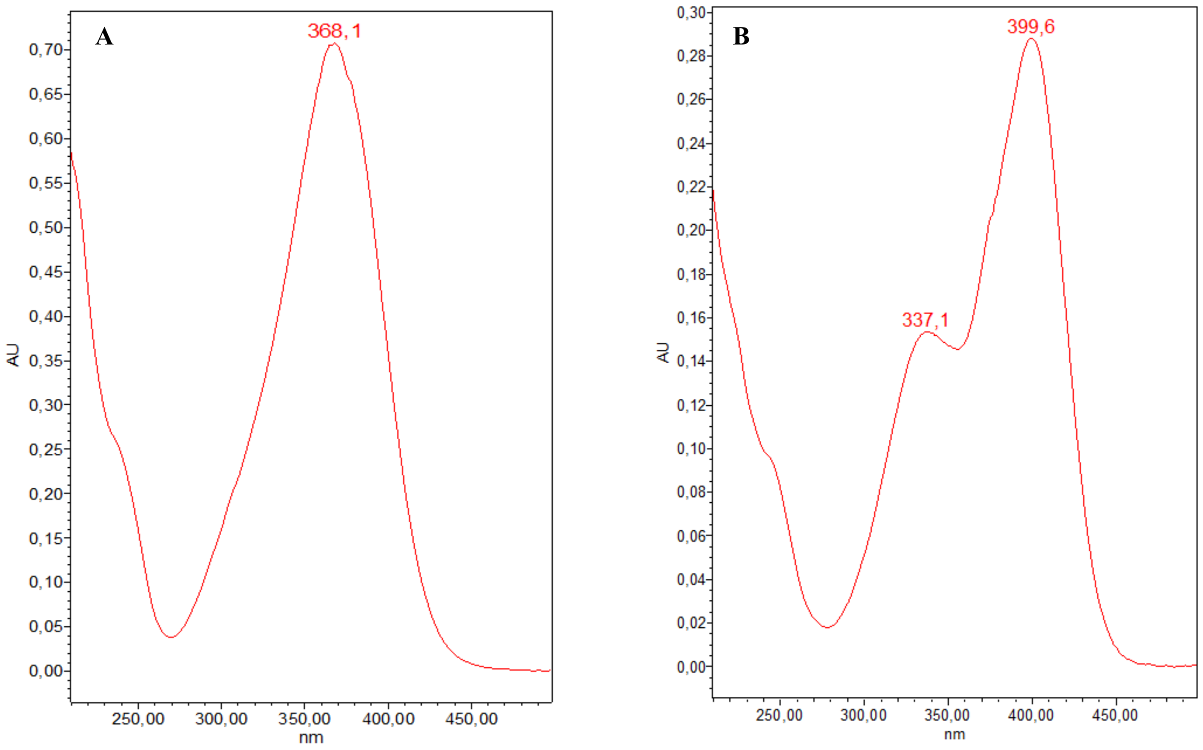

Figure S3. *HRMS spectrum of (Z)-6,4'-dihydroxy-4-methoxy-7-prenylaurone (**2**)*

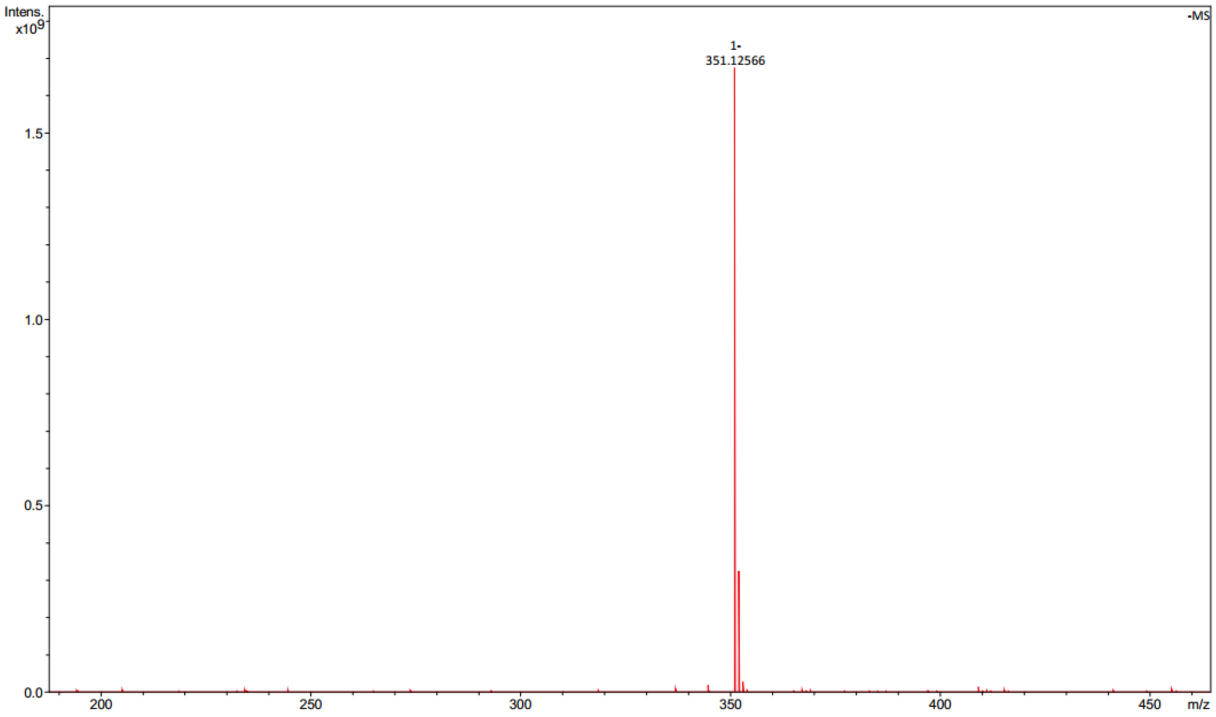

Figure S4. *^{1}H NMR spectra of:* ***A*** *xanthohumol (**1**),* ***B*** *its aurone (**2**)*

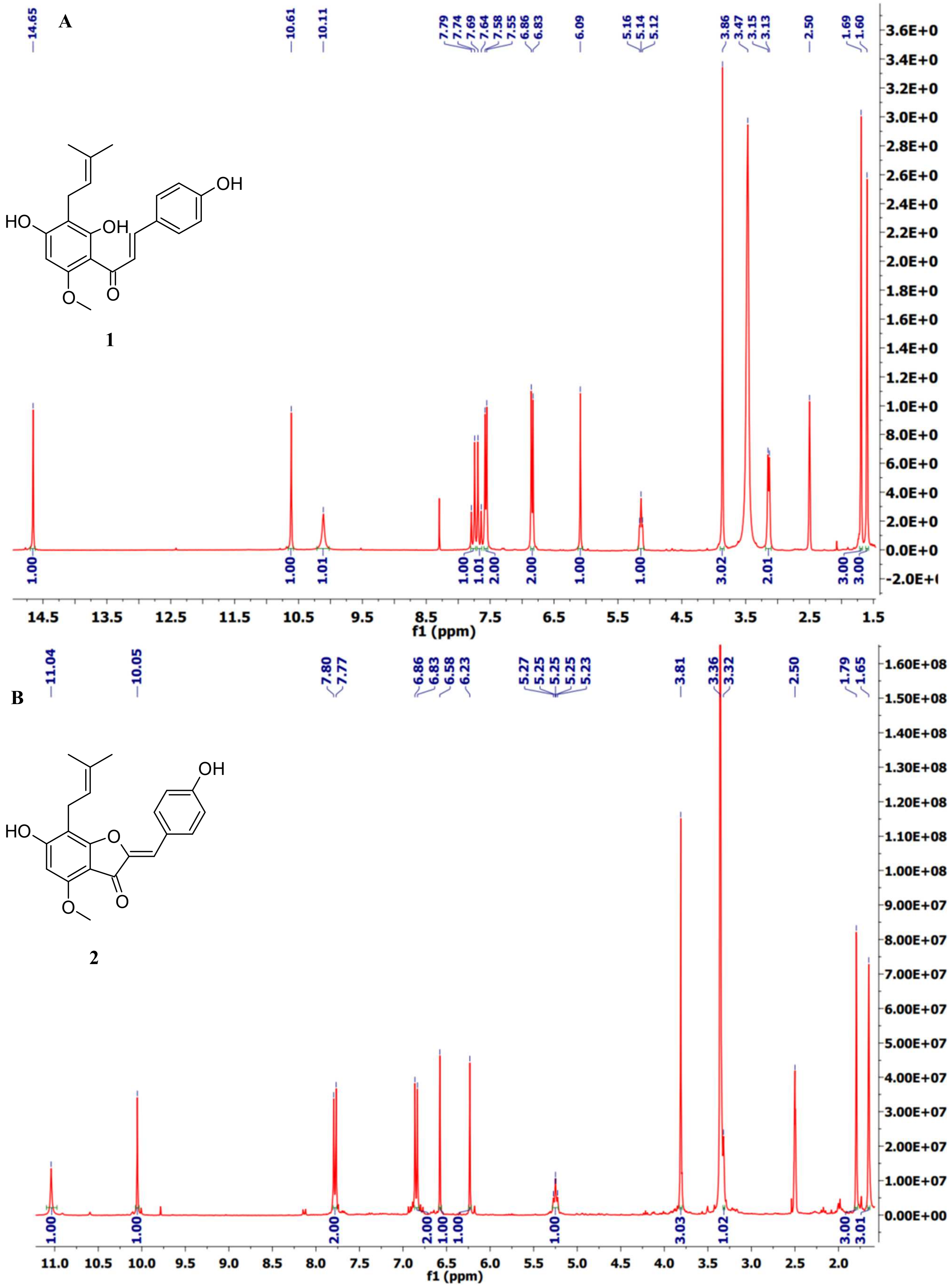

Figure S5. *^{13}C NMR spectra of:* ***A*** *xanthohumol (**1**),* ***B*** *its aurone (**2**)*

A

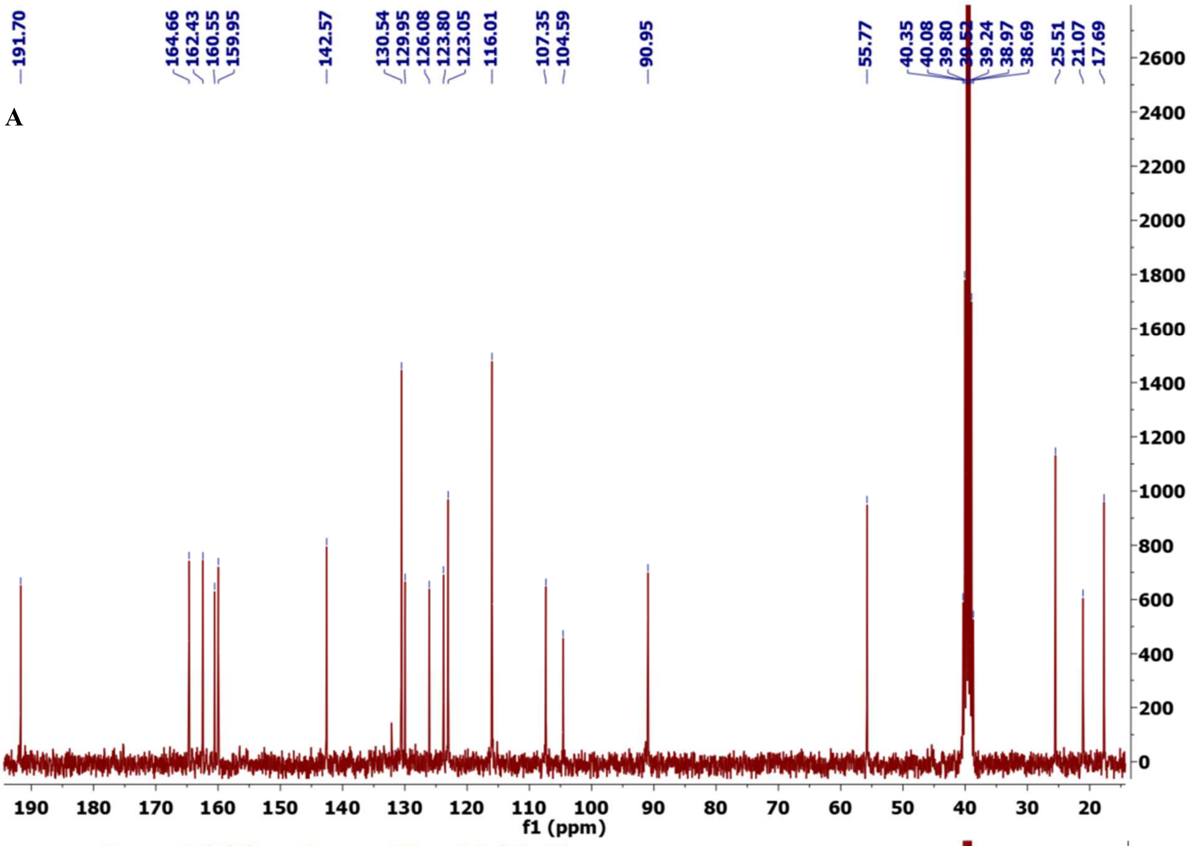

B

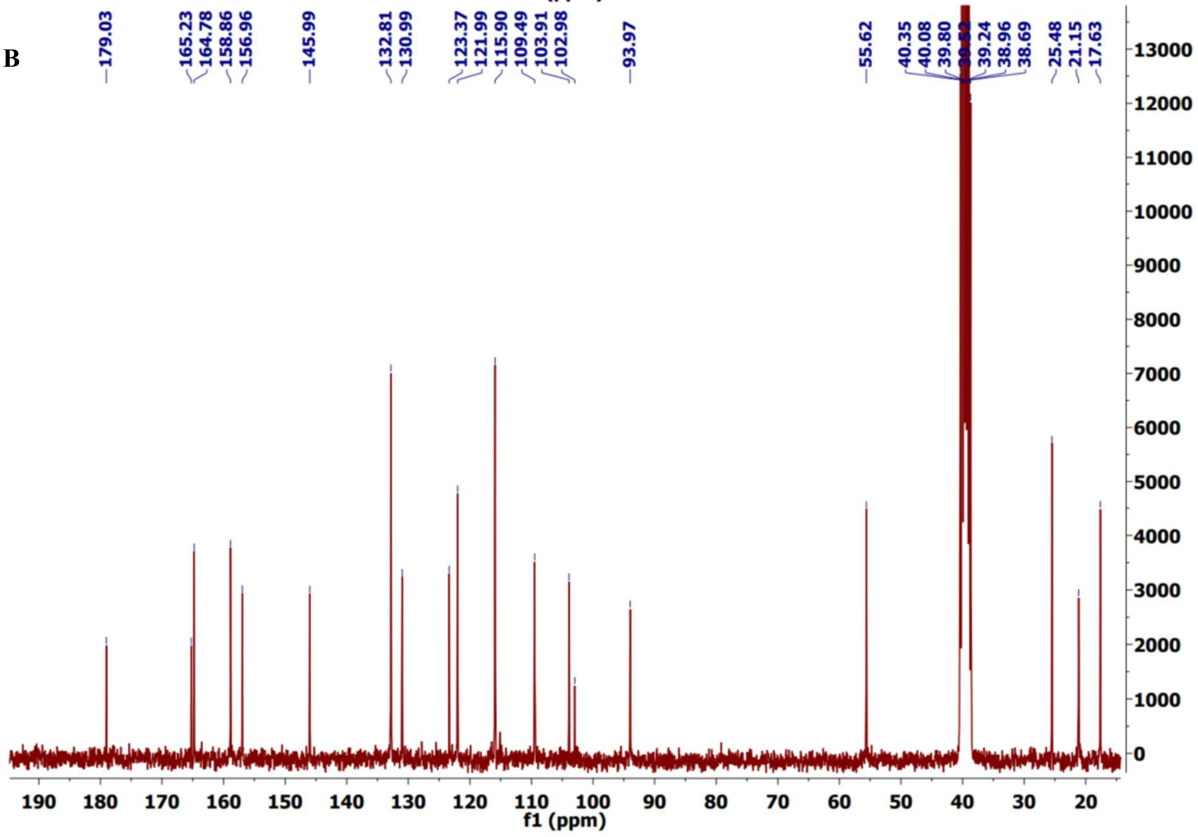

Figure S6. *^{1}H-^{1}H NMR (COSY) spectrum of (Z)-6,4'-dihydroxy-4-methoxy-7-prenylaurone (**2**)*

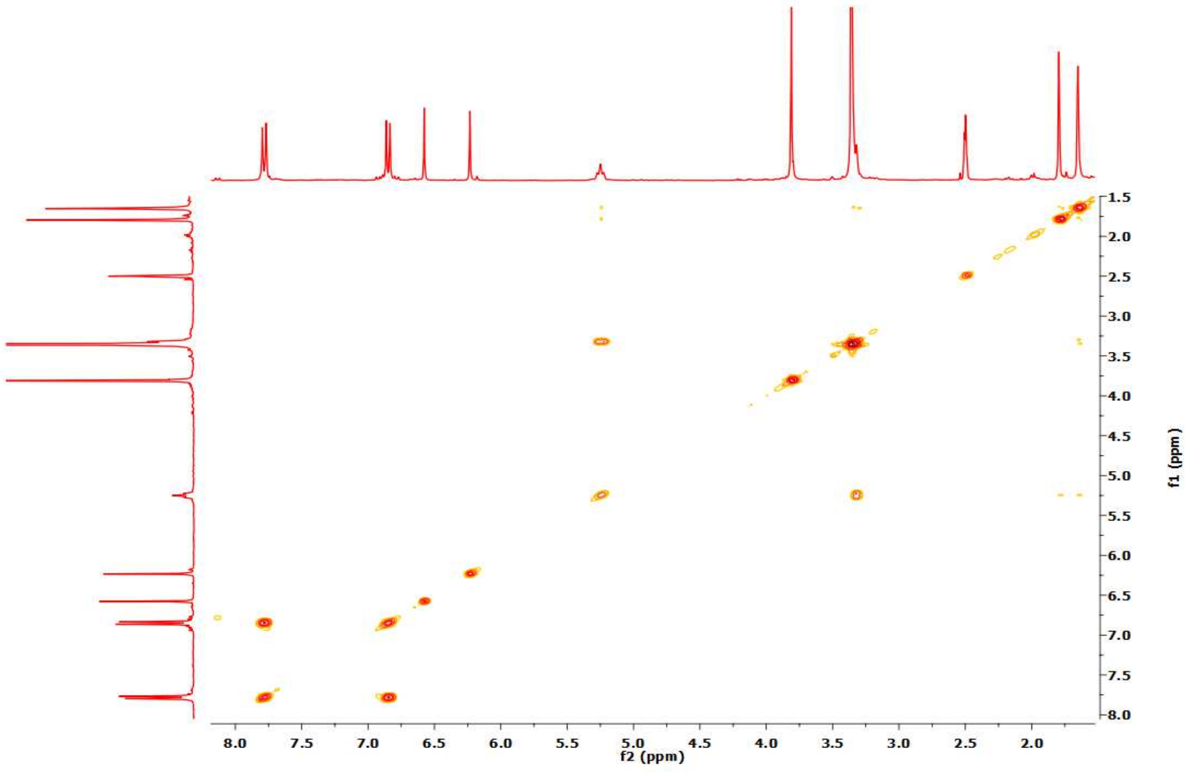

Figure S7. *^{1}H– ^{13}C NMR (HSQC) spectrum of (Z)-6,4'-dihydroxy-4-methoxy-7-prenylaurone (**2**)*

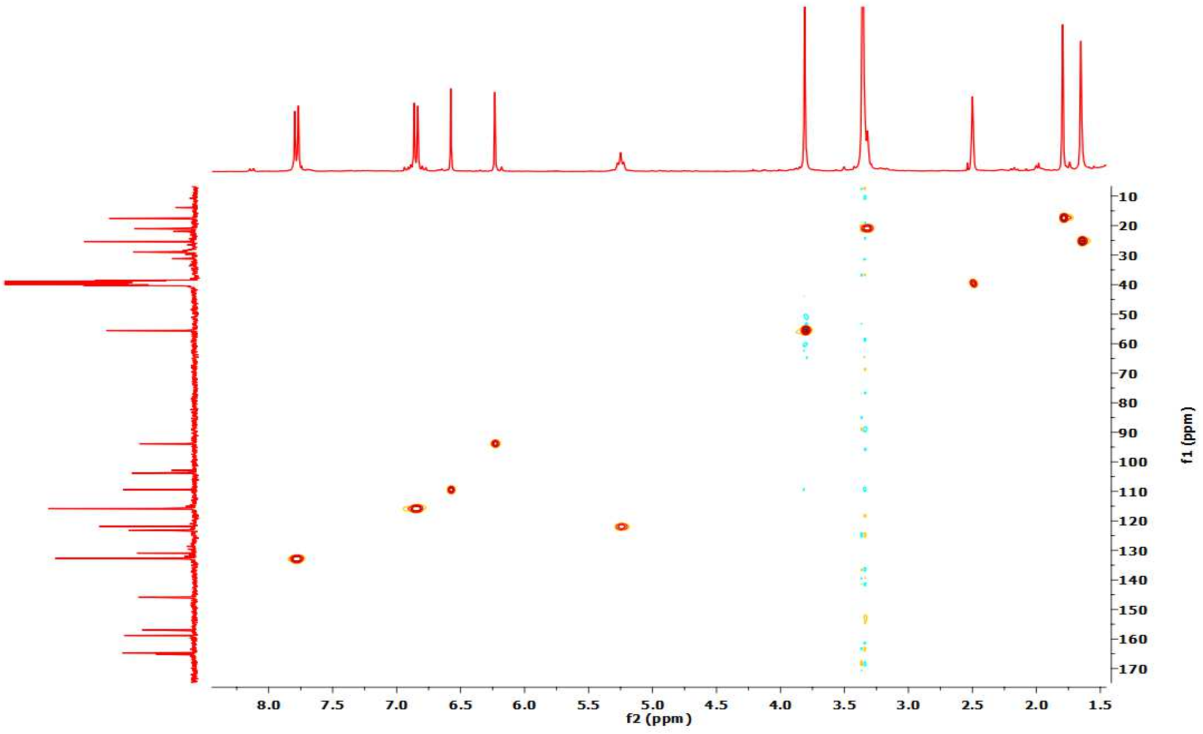

Table S1. *In vitro antiproliferative activity of xanthohumol (**1**) and aurone (**2**) against human cancer and normal human and murine cell lines.*

	Cell line	Antiproliferative activity IC_{50} [a] (µM)		
		**1**	**2**	Cisplatin (reference)
Cancer	MCF-7	9.29 ± 0.27	9.28 ± 0.50	11.30 ± 0.99
	SK-BR-3	12.57 ± 2.28	11.31 ± 0.17	21.57 ± 3.93
	T47D	8.27 ± 1.23	8.09 ± 2.21	13.08 ± 5.58
	HT-29	91.31 ± 8.92	62.09 ± 16.52	18.95 ± 2.25
	LoVo	5.53 ± 1.64	8.85 ± 4.85	4.32 ± 0.90
	LoVo/Dx	7.28 ± 1.22	7.83 ± 2.82	8.97 ± 1.45
	PC-3	9.15 ± 0.88	19.06 ± 9.27	15.29 ± 2.28
	Du145	10.34 ± 0.42	14.71 ± 4.42	9.36 ± 0.63
	A549	10.02 ± 0.71	16.59 ± 3.29	11.34 ± 1.36
	MV-4-11	8.07 ± 0.52	7.45 ± 0.87	1.11 ± 0.36
Normal	HLMEC	36.30 ± 6.70	52.82 ± 8.03	1.26 ± 0.1
	BALB3T3	19.62 ± 2.95	44.46 ± 5.16	8.96 ± 2.15

[a] Data represent the mean values of at least three independent experiments (n = 3) ± SD

Table S2. *The selectivity index (SI) which represents IC_{50} for normal cell line/IC_{50} for cancerous cell line*

Cell line	Calculated selectivity index (SI)*					
	**1**		**2**		Cisplatin	
	SI_A	SI_B	SI_A	SI_B	SI_A	SI_B
MCF-7	3.91	2.11	5.69	4.79	0.11	0.79
SK-BR-3	2.89	1.56	4.67	3.93	0.06	0.42
T47D	4.39	2.37	6.53	5.50	0.10	0.69
HT-29	0.40	0.21	0.85	0.72	0.07	0.47
LoVo	6.56	3.55	5.97	5.02	0.29	2.07
LoVo/Dx	4.99	2.70	6.75	5.68	0.14	1.00
PC-3	3.97	2.14	2.77	2.33	0.08	0.59
Du145	3.51	1.90	3.59	3.02	0.13	0.96
A549	3.62	1.96	3.18	2.68	0.11	0.79
MV-4-11	4.50	2.43	7.09	5.97	1.14	8.07

* Selectivity index (SI) was calculated for each compound using the following formula: SI_A = IC_{50} for normal cell line (HLMEC)/IC_{50} for respective cancerous cell line as indicated on each plot; SI_B = IC_{50} for normal cell line (BALB/3T3)/IC_{50} for respective cancerous cell line as indicated on each plot. SI >1.0 indicates a drug with the efficacy against tumor cells greater than the toxicity towards normal cells. SI < 1.0 non-selective action.

References:

1. Vogel, S.; Ohmayer, S.; Brunner, G.; Heilmann, J., Natural and non-natural prenylated chalcones: Synthesis, cytotoxicity and anti-oxidative activity. *Bioorganic & Medicinal Chemistry* **2008,** 16, (8), 4286-4293.
2. Nookandeh, A.; Frank, N.; Steiner, F.; Ellinger, R.; Schneider, B.; Gerhäuser, C.; Becker, H., Xanthohumol metabolites in faeces of rats. *Phytochemistry* **2004,** 65, (5), 561-570.
3. Khupse, R.; Erhardt, P., Total synthesis of xanthohumol. *Journal of Natural Products* **2007,** 70, 1507 - 1509.
4. Tronina, T.; Strugała, P.; Popłoński, J.; Włoch, A.; Sordon, S.; Bartmańska, A.; Huszcza, E., The influence of glycosylation of natural and synthetic prenylated flavonoids on binding to human serum albumin and inhibition of cyclooxygenases COX-1 and COX-2. *Molecules* **2017,** 22, (7), 1230.
5. Stevens, J. F.; Miranda, C. L.; Frei, B.; Buhler, D. R., Inhibition of peroxynitrite-mediated LDL oxidation by prenylated flavonoids: The α,β-unsaturated keto functionality of 2'-hydroxychalcones as a novel antioxidant pharmacophore. *Chemical Research in Toxicology* **2003,** 16, (10), 1277-1286.
6. Shanker, N.; Dilek, O.; Mukherjee, K.; McGee, D.; Bane, S., Aurones: Small Molecule Visible Range Fluorescent Probes Suitable for Biomacromolecules. *J Fluoresc* **2011,** 21, (6), 2173-2184.
